# Institutional Needs

**Published:** 1917-05-05

**Authors:** 


### Institutional Needs.

### A FORMALDEHYDE VAPORISER FOR USE WITH A STEAM DISINFECTOR.

(Manlove, Alliott & Co., Ltd., Bloomsgrove Works, Nottingham.)

This patent is the invention of two medical officers of health, Dr. T. S. Higgins, of London, and Dr. J. Dale, of Birmingham. The apparatus consists of a copper vessel of about three gallons net capacity, in which a mixture of formaldehyde and water is placed. The mixture is vaporised by a steam circulating nozzle in the generator, and the vapour enters the interior of the disinfector chamber by means of a connection between the hot-air apparatus and the disinfector. The generation of the vapour takes place at a comparatively low temperature, corresponding to the degree of vacuum in the generator. Thus the use of this vaporiser with a steam disinfector enables delicate materials and fabrics, which would be damaged by high-pressure steam, to be disinfected at a low temperature by formaldehyde vapour without sustaining injury or damage.

### PHENALGIN TABLETS.

We have received from Messrs. E. T. Pearson and Co., Mitcham, Surrey, samples of the above. As is well known, it consists of a stimulant form of an excellent analgæsic and anti-neuralgic base. This combination enables the prescriber to be much more certain of obtaining relief without depression. We have ourselves made use of the preparation for many years, especially in cases of headache and facial neuralgia, and have found it to be both useful and trustworthy. It can also be given successfully in painful menstruation and in slight cases of insomnia.

### MODERN GRATES.

Our attention has been called to the wording of a notice of labour-saving appliances, under this heading, which appeared on page 56 of The Hospital for the 21st ultimo, and to the illustrations. Those illustrations represent the "Bewty" Front and Fire, made solely by the Interoven Stove Company, 156 Charing Cross Road. The "Bewty" Fire Adjustable Barless Fronts (Pascall's Patent) of the Interoven Stove Company are claimed to be the most effective, well-constructed, and durable in the market. It is due to the Interoven Stove Company and to our readers that they should realise the precise facts in regard to the "Bewty" Fire which we have here the satisfaction to make clear.

